# 3D porous nanostructured Ni_3_N–Co_3_N as a robust electrode material for glucose fuel cell

**DOI:** 10.1039/c9ra08812a

**Published:** 2020-02-11

**Authors:** Muhammad Irfan, Izhar Ullah Khan, Jiao Wang, Yang Li, Xianhua Liu

**Affiliations:** Tianjin Key Lab. of Indoor Air Environmental Quality Control, School of Environmental Science and Engineering, Tianjin University Tianjin 300354 PR China lxh@tju.edu.cn

## Abstract

Metal nitrides are broadly applicable in the field of electrochemistry due to their excellent electrical properties. In this study, a 3D nanostructured Ni_3_N–Co_3_N catalyst was prepared by using a versatile urea glass method, and was tested as an anode catalyst for a glucose fuel cell. The synthesized Ni_3_N–Co_3_N exhibits uniform particle dispersion in structure, morphology, and composition, and has a interpenetrating three-dimensional network structure. Notably, the Ni_3_N–Co_3_N significantly improved the catalytic activity of glucose oxidation compared to Ni_3_N, Co_3_N, and conventional activated carbon electrodes. The superior electrochemical performance could be attributed to its porous structure and unique properties, which provided a fast transport network for charge and mass transfer as well as good synergetic effect. The glucose fuel cell equipped with a Ni_3_N–Co_3_N anode achieved 30.89 W m^−2^ power and 97.66 A m^−2^ current densities at room temperature. This investigation provides potential directions for the design of cost-effective bimetallic catalysts for a wide range of glucose fuel cell applications.

## Abstract 2

Metal nitrides are broadly applicable in the field of electrochemistry due to their excellent electrical properties. 3D nanostructured Ni_3_N–Co_3_N catalyst was prepared and tested as anode catalyst for a glucose fuel cell.

## Graphical abstract

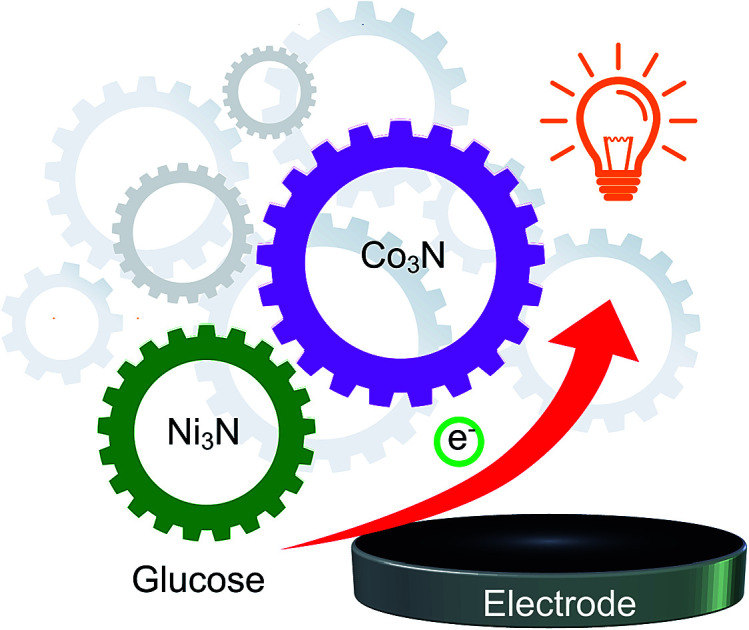

## 1. Introduction

Global energy demand has been accelerating at an alarming rate due to industrialization, and rapid population growth.^1^ In order to overcome the disaster triggered by energy depletion, renewable energy demand has gained great attention worldwide. For efficient renewable energy utilization, it is important to develop high performance, cost-effective, environmentally benign energy conversion and storage systems. Among the various possibilities, fuel cells represent the typical electrochemical energy conversion technologies, which could afford high power density, high energy density and long-term stability.^2^ Hydrogen fuel cells are considered one of the most promising power sources, due to their high efficiency and low emissions.^3^ However, hydrogen is very flammable and difficult to store. The use of glucose as a hydrogen carrier in fuel cells would not only offer ease of storage and transportation but may also pave the way for a carbon-neutral future. Glucose is non-toxic, abundantly available, and can easily be derived from waste biomass.^2^ In addition, glucose has the capability to release 2870 kJ mol^−1^ energy after fully oxidizing to carbon dioxide and water by transferring its 24 electrons, theoretically.^4^ These factors make glucose an attractive fuel from both economic and environmental perspectives.^5,6^

Despite the significant interest from scientists in developing glucose fuel cell technology, the direct utilization of glucose in fuel cells is largely underdeveloped due to the sluggish glucose oxidation reaction.^7–9^ Thus, a significant effort is required in developing low-cost and highly efficient anode catalysts which can facilitate glucose oxidation. Platinum-based metals have been commonly employed as anode catalysts in fuel cells due to their low over-potentials and fast kinetics in the redox reactions.^10,11^ However, high cost, resource limitation, and easily poisoned sensitivity hamper their wide applications.^12^ Scientists are working to find substitutes of noble metals by improving the properties of transition metals such as (Ni, Co, Fe, Cu, and Zn). Various types of active substances, such as transition metals and their oxides/hydroxides, are frequently used in fuel cells,^13^ supercapacitors,^14^ batteries,^15^ glucose sensors^16,17^*etc.* as electrode materials owing to their unique layered structure and chemical stability. Among these materials, metallic nickel, nickel alloys and nickel compounds are preferred because of their low cost, good electrochemical stability, resistance to poisoning and high catalytic activity in alkaline environment.^18^ They are also widely used as bimetallic catalysts for water splitting,^19^ oxygen reduction reaction (ORR),^20,21^ ethanol,^22,23^ hydrazine,^24^ methanol^25^ and glucose oxidation.^13^ Nickel and Cobalt oxides are considered propitious transition metal oxide combinations used for batteries,^26^ fuel cell^13^ and supercapacitors.^15,27,28^ Gao *et al.*,^13^ used Ni–Co composite catalyst in a direct glucose fuel cell and obtained 23.97 W m^−2^ peak power density. Similarly, Fen *et al.*,^29^ and Jing *et al.*,^30^ used bimetallic nickel–cobalt catalyst for urea hydrogen peroxide and methanol fuel cell. These studies demonstrated interesting electrochemical properties of nickel and cobalt, indicating their potentials as electrode materials. The cost-effective association of these two metals has higher electrochemical activity in binary metal oxide attributed to the multiple redox mechanism and the synergistic effects in fuel cells.^13,31^

Transition metal nitrides are another type of material that exhibits excellent conductance, good stability and speeds up charge transport when employed as electrode.^32–34^ On the other hand, the presence of nitrogen strongly influences the electronic properties of the metal by increasing the density of electrons on the surface of the metal. Therefore, the metal nitride has a higher electro-catalytic activity in the reduction reaction than the corresponding pure metal.

The urea-glass route is a carbothermal reduction method for the synthesis of various metal carbides and nitrides in the presence of an N/C source.^35^ It has the advantages of being simple, scalable, and versatile. A key feature of the “urea glass technology” is the formation of a gel-like starting material consisting of a polymer composite between a metal precursor and urea, and environmental treatment of the corresponding carbides and nitrides.^22^ This is an easy way to minimize the use of toxic solvents and does not require purification. In fact, this is a simple and safe way to use urea as a nitrating agent instead of high-pressure ammonia to produce nitrides.

In this study, a low-cost and efficient nickel–cobalt nitride (Ni_3_N–Co_3_N) composite with 3D-porous nanostructure was prepared by using a facile urea glass method. That prepared composite was applied for direct glucose oxidation in the simple and non-toxic method. The performance results demonstrated that Ni_3_N–Co_3_N nanoparticles are an effective platform for the electrooxidation of glucose in an alkaline medium. The formation of Ni_3_N–Co_3_N was confirmed by XRD, SEM and XPS techniques. Our results showed that nickel–cobalt nitride composite has good electrocatalytic activity and is a promising fuel cell catalyst.

## 2. Methods

### 2.1 Synthesis of Ni_3_N–Co_3_N catalyst

All materials are used without further refining. Co(NO_3_)_2_·6H_2_O, Ni(NO_3_)_2_·6H_2_O, urea, glucose, ethanol, activated carbon (AC), PTFE (Hesen Inc. Shanghai, China) solution, Nafion (5% wt), and KOH were all analytical grades. For the preparation of Ni_3_N–Co_3_N composite catalyst, Co(NO_3_)_2_·6H_2_O (0.8730 g), Ni(NO_3_)_2_·6H_2_O (0.8720 g) and 0.414 g urea (H_2_NCONH_2_) were dissolved in 6.0 mL ethanol solution. The resultant mixture was stirred magnetically for 12 hours. The sample was then heat-treated for 5 h in a tube furnace at 350 °C under a nitrogen (N_2_) flowing atmosphere. For comparison, Ni_3_N and Co_3_N were also synthesized separately by using half the concentration of urea.

### 2.2 Physical characterization

The synthesized catalysts were physically characterized by X-ray diffraction (XRD, Brucker D8) and scanning electron microscopy (SEM, Hitachi S4800, Japan). X-ray photoelectron spectroscopy (XPS) spectra of materials were obtained on a PHI 5000C ESCAESCALAB 250 spectrometer using a monochromatism Al Kα (1486.6 eV) to examine the valence of materials and surface species.

### 2.3 Electrochemical characterization

Electrochemical analysis of synthesized catalysts was carried out at ambient temperature on an electrochemical workstation (CHI 660E, CHI Instrument Co. Ltd, Shanghai, China) with a typical three-electrode system. The catalyst inks of Ni_3_N, Co_3_N, and Ni_3_N–Co_3_N were prepared separately for cyclic voltammetry (CV), linear sweep voltammetry (LSV) and electrochemical impedance spectroscopy (EIS) measurements as follow: 10 mg of catalyst powder was mixed in 1 mL solution (20 μL of 5% Nafion solution, 653 μL pure water, 327 μL ethanol) and then ultra-sonicated for 30 min to form homogenous solution. Further for energy and power density, a direct glucose alkaline fuel cell was assembled. The fabrication of anode for a fuel cell based on the following procedure, a mixture of 85% by weight of activated carbon (AC), 10% by weight of PTFE, and 5% by weight of Ni_3_N–Co_3_N composite catalyst dissolved in ethanol solution followed by sonication. A black mud was obtained after dying by a water bath and pressed on nickel foam followed by our previous reports.^7–9,36–38^ While, Ni_3_N, Co_3_N, and AC (without catalyst) anodes were prepared with the same configuration. A similar air cathode^38,39^ used in all fuel cells.

## 3. Results and discussion

### 3.1 Material characterization of Ni_3_N–Co_3_N

**Fig. 1:**
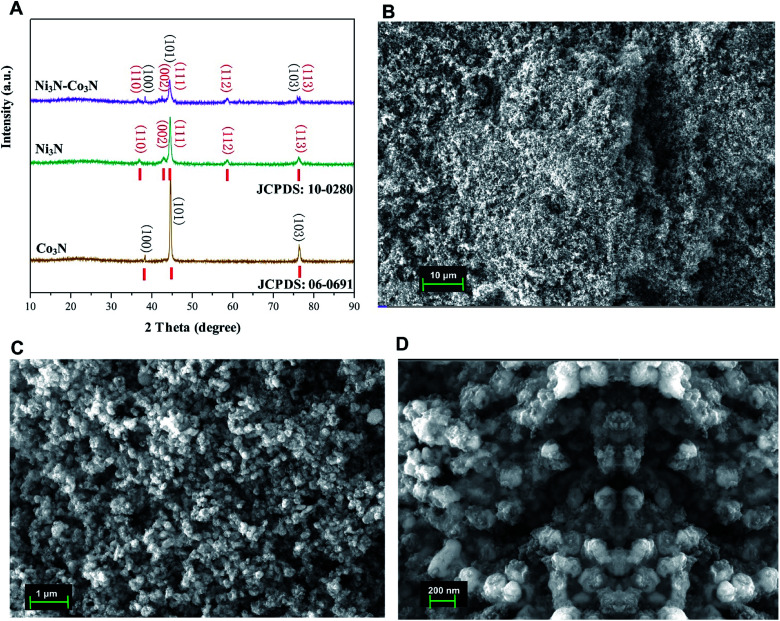
(A) X-ray diffraction (XRD) patterns of Ni_3_N, Co_3_N, and Ni_3_N–Co_3_N; (B–D) SEM images of Ni_3_N–Co_3_N at 10 μm, 1 μm, and 200 nm resolution.

The XRD pattern of the nitrogenated materials is shown in Fig. 1A. The diffraction peaks could be well pointed to orthorhombic Co_3_N phase (JCPDS no. 06-0691) and hexagonal Ni_3_N phase (JCPDS no. 10-0280). All the peaks correspond to the (100, 101, 103) of Co_3_N and (110, 002, 111, 112, 113) of Ni_3_N, respectively.^40^ The XRD spectra of Co_3_N and Ni_3_N demonstrate a similar pattern due to the size and valence of Ni and Co atoms are similar. XRD pattern of Ni_3_N–Co_3_N is like that of the single metal counterpart, except minor peak shifts due to alloying of nickel and cobalt.

The morphology of Ni_3_N–Co_3_N nanoparticles can be observed by SEM images which is illustrate in Fig. 1B–D, that showed the agglomeration of particles, with particle size on the nanometer scale. At 1 μm-magnification SEM image (Fig. 1C), the composite exhibits a similar network structure and abundant interconnected channels, which significantly contributes to the diffusion and transfer of ions from the bulk solution to the inner surface of the porous material. Fig. 1D reveals that roughly spherical Ni_3_N–Co_3_N nanoparticles having a diameter of about 20 nm are agglomerated with smaller nanoparticles. Thus, it can be inferred that the particle size of the Ni_3_N–Co_3_N catalyst depends on the degree of agglomeration between the smaller particles. The small dots on agglomerated particles can bring a beneficial effect on substrate contact through increasing the surface area of the composite.

**Fig. 2:**
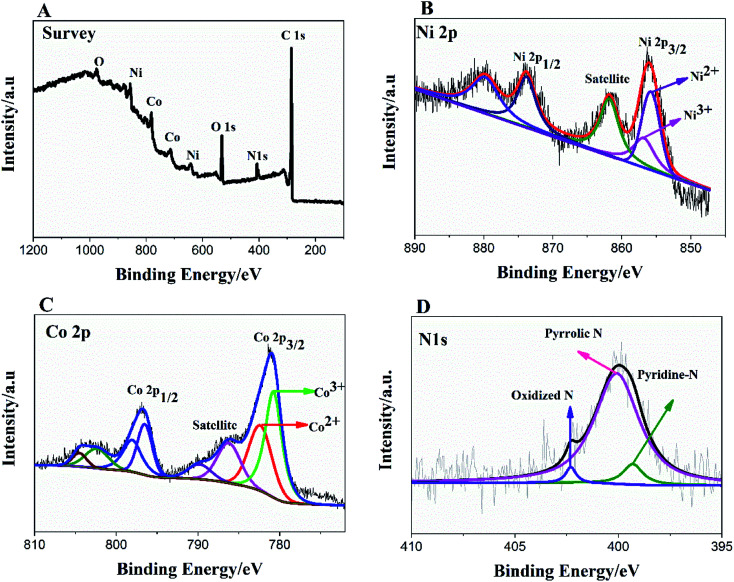
XPS survey spectrum of Ni_3_N–Co_3_N (A); high resolution of Ni 2p (B), Co 2p (C) and N 1s spectra (D).

**Fig. 3:**
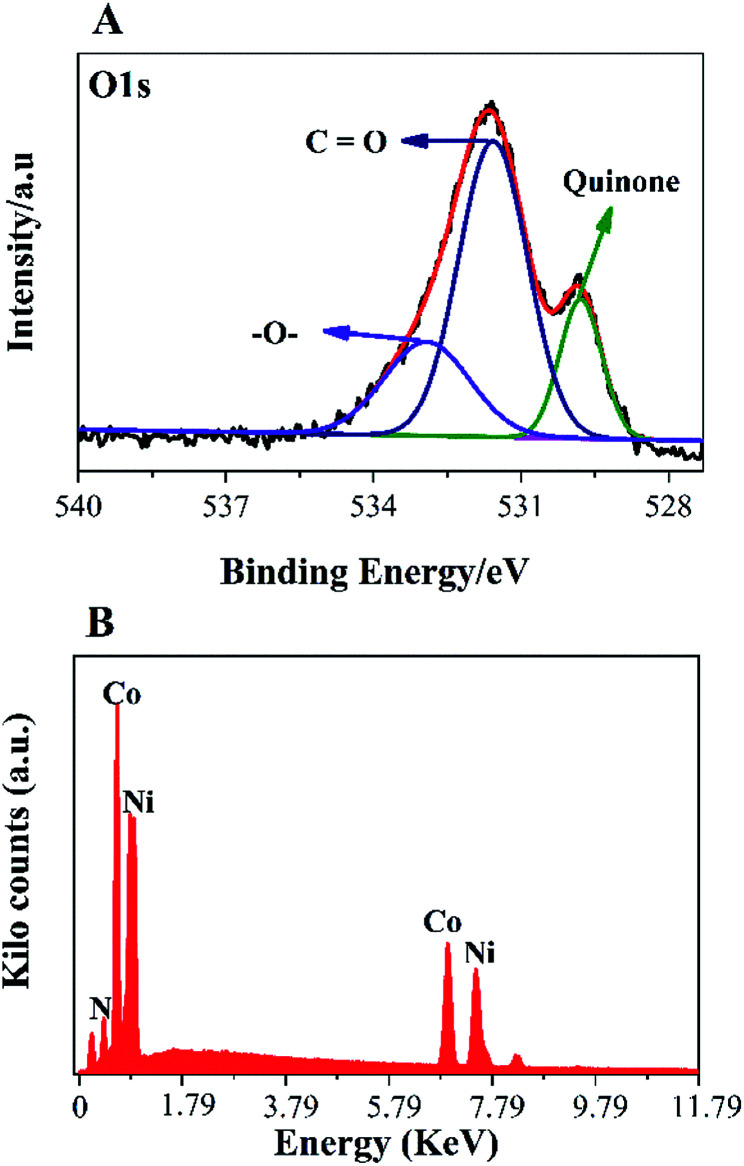
(A) O1s spectra; EDX spectra (B) of the Ni_3_N–Co_3_N bimetallic catalyst.

To further, study the electronic states of Ni_3_N–Co_3_N, X-ray photoelectron spectroscopy (XPS) measurements were performed. The survey spectrum (Fig. 2A) indicates the existence of C 1s, O 1s, N 1s, Ni and Co. The O 1s peaks slighter shift towards higher binding energy due to the Ni and Co attachment. Narrow range Ni 2p spectra is provided in Fig. 2B, two peaks observed at a binding energy of 856.01 eV and 855.45 eV which indicates the presence of Ni^3+^ and Ni^2+^ (ref. 40 and 41) respectively. In Ni 2p spectrum, the peak of nickel nitride shifts to higher binding energy, which is probably due to the nickel atoms are surrounded by Co atoms and having fewer electrons. Therefore, the decrease in the electron shielding effect causes the positive shift of the Ni 2p peak, which confirmed the peak shift in XRD results due to alloying of nickel and cobalt. Fig. 2C, indicates that cobalt is present in the oxidation states of Co^3+^ (780.68 eV) and Co^2+^ (782.06 eV), similar to the literature results for N atom coordinated Co^3+^ and Co^2+^.^41^ The N 1s spectrum has three peaks: pyridine-N (398.4 ± 0.2 eV), pyrrolic N (400.2 eV), and oxidized N (402.3 eV) (Fig. 2D).^42^ Recent studies have shown that pyridine-N and pyrrolic N act as effective chemically active sites for the dyadic response. In addition, graphite N may contribute to electron transport, while oxidized N improves surface wettability and facilitates the transport of ions from electrolyte solutions to the interface.^43^ In the fitting of O 1s XPS spectrum (Fig. 3A), the composite sample showed three peaks at 530.6, 531.5, 533.2 and 537.7 eV, respectively. The peak value of 530.6 eV corresponds to quinone. The peak at 531.5 eV is due to C=O in the carboxyl (COOH) and/or carbonyl (C=O) groups. The fitted peak at binding energy = 533.2 eV represents single bond oxygen (–O–). Moreover, these oxygen-containing groups not only enhance the wettability, but also reduce the internal resistance, and can also reversibly react with hydrogen ions under alkaline conditions, and the electrochemical performance and catalytic ability can be greatly improved. According to the above analysis, the functional groups of the heteroatoms have been successfully bonded to the basal or graphite lattice edges, and due to good wettability, can contribute to glucose oxidation, and enhanced conductivity as well as low ion diffusion/transport. Further confirmation of the XPX spectrum, energy-dispersive X-ray spectroscopy (EDX) was performed (Fig. 3B). The EDX spectra represent the occurrence of Ni, Co, and N which is consistent with Co 2p, Ni 2p, and N 1s respectively.

### 3.2 Electrochemical oxidation of glucose at Ni_3_N–Co_3_N

**Fig. 4:**
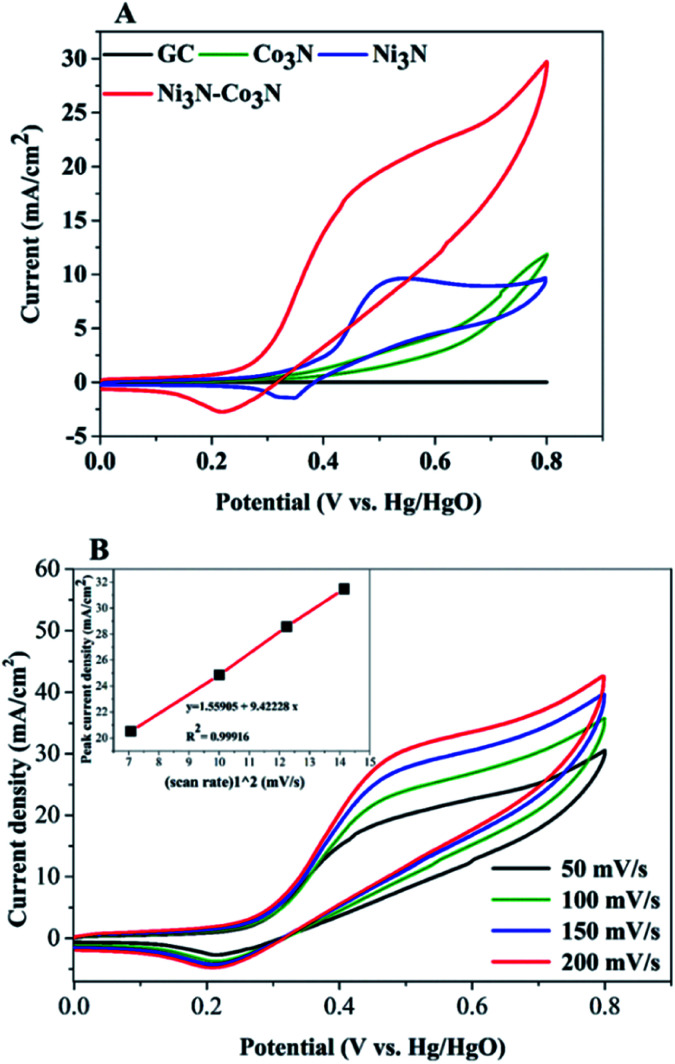
(A) CV curves of Ni_3_N, Co_3_N and Ni_3_N–Co_3_N at 50 mV s^−1^ scan rate; (B) CV curves of Ni_3_N–Co_3_N scanned at different scan rates (50–200 mV s^−1^), (inset) square root of scan root *vs.* peak current density mA cm^−2^. Condition: 1 M glucose and 3 M KOH.

**Fig. 5:**
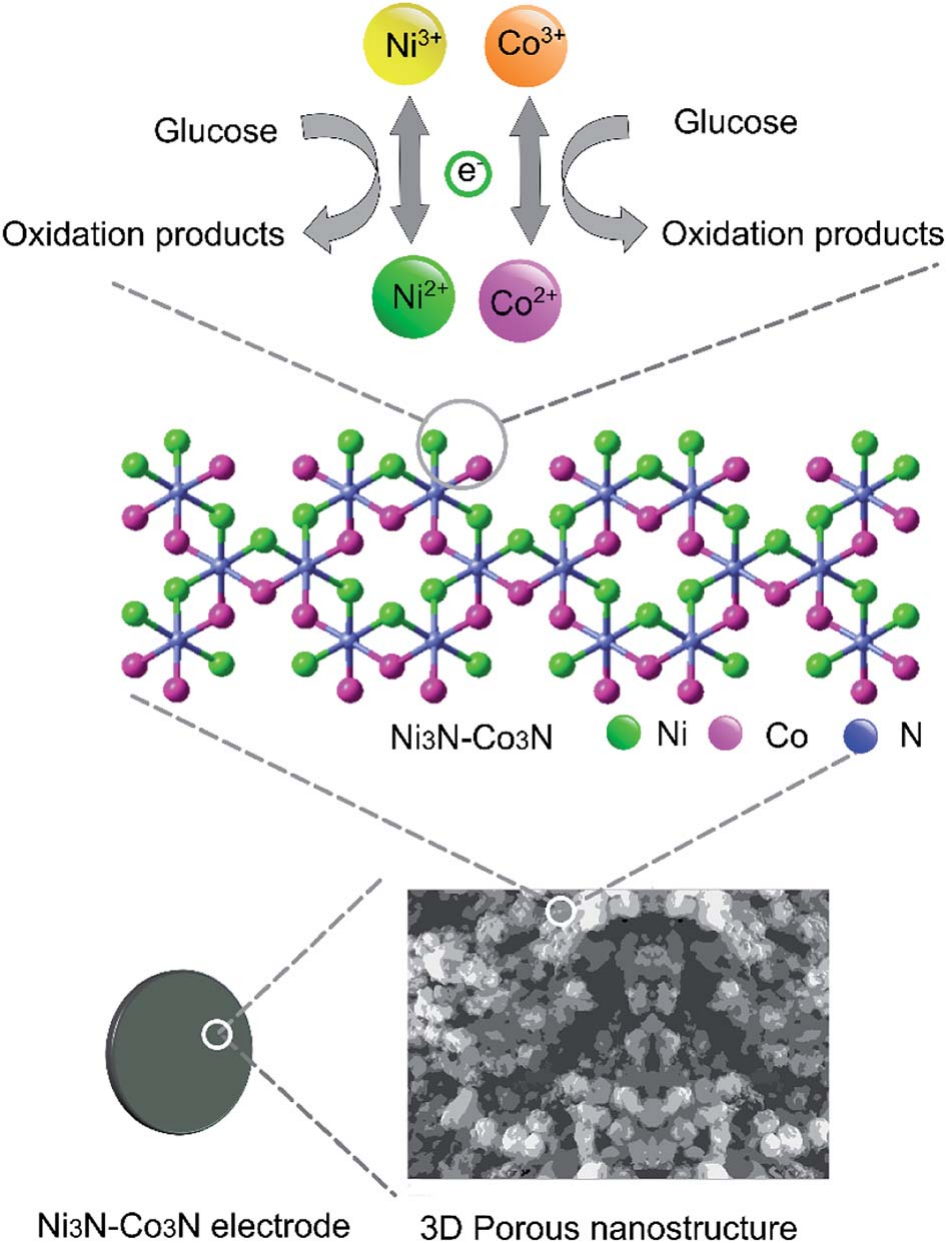
Schematic representation of Ni_3_N–Co_3_N modified electrode and proposed acting mechanism of Ni_3_N–Co_3_N catalyst in a glucose fuel cell.

In order to examine electrochemical properties as prepared catalysts in an alkaline environment, the catalyst ink was deposited by drop-casting on a glassy carbon electrode to evaluate CV at 50 mV s^−1^ scan rate within 0–0.8 V potential range (Fig. 4A). Electrochemical responses of bare GC, Co_3_N, Ni_3_N, and Ni_3_N–Co_3_N electrodes were compared in 3 M KOH and 1 M glucose solution. The CV behaviour of bare GCE did not respond to glucose oxidation. After modification of the GC electrode with Co_3_N, Ni_3_N, and Ni_3_N–Co_3_N nanoparticles, new peaks appeared which can be ascribed to the glucose electro-oxidation. The catalytic current for glucose oxidation should be due to the removal of hydrogen and chemisorption at hemiacetalic (C1) carbon.^13^ The oxidation and reduction peaks of Ni_3_N appeared between 0.25 to 0.65 V (*vs.* Hg/HgO). These peaks are regarded as the reversible conversion of Ni^2+^ ↔ Ni^3+^ in the alkaline system, it means that the glucose is possibly electrooxidize on Ni^3+^ and release electron. The electric current of Ni_3_N–Co_3_N was higher than Ni_3_N and Co_3_N, while the onset potential of Ni_3_N–Co_3_N is 0.16 V, it indicates that the decline in onset potential is due to the generation of Ni^3+^ at smaller potential. The onset potential remains same at various scan rates from 50 mV s^−1^ to 200 mV s^−1^ (Fig. 4B), while the current increased by increasing scan rate and there is a linear relationship exist between the anodic current and the square root of the scan rate, indicate that the electrochemical behaviour of Ni_3_N–Co_3_N for glucose oxidation is a diffusion-controlled process. The modification of Ni_3_N with Co_3_N gets the benefit of reducing onset potential that promotes the Ni^3+^ formation at smaller potential. The possible electro-catalytic mechanism involved in glucose oxidation is illustrated in Fig. 5. In the alkaline medium, the Ni_3_N–Co_3_N anode would be following the similar redox mechanism as the NiO and CoO.^44,45^ The purposed chemical reactions involved in glucose oxidation catalysed by Ni and Co under alkaline conditions can be represented by the following equations:

1 Ni^2+^ + OH^−^ ↔ Ni^3+^ + e^−^

2 Co^2+^ + OH^−^ ↔ Co^3+^ + e^−^

3 Ni^3+^ + glucose → Ni^2+^ + glucolactone

4 Co^3+^ + glucose → Co^2+^ + glucolactone

5 Ni_3_Co_3_ + glucose → Ni_2_Co_2_ + glucolactone

Two redox couples (Ni^3+^/Ni^2+^ and Co^3+^/Co^2+^) may play important roles in this process. In an alkaline condition, both Ni^2+^ and Co^2+^ can be transformed into Ni^3+^ and Co^3+^, respectively, and give electrons to the current collector. The formed Ni^3+^ and Co^3+^ can transform glucose into oxidation products and regenerate Ni^2+^ and Co^2+^. There may exist a synergic effect between the two redox couples. It has been proposed that nickel can effectively catalyze the glucose oxidation and cobalt can facilitate the transportation of reaction products produced from glucose oxidation.^13^ Furthermore, the 3D porous nanostructure of Ni_3_N–Co_3_N can benefit the uniform dispersion of catalytically active sites on the composite surface.

**Fig. 6:**
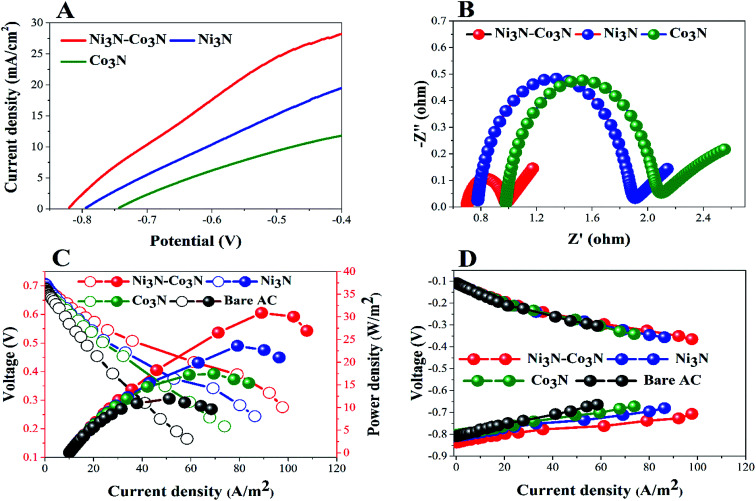
(A) LSV and EIS (B); power density (C) and polarization curves (D) of glucose fuel cells.

In order to determine the substantial responses of Ni_3_N, Co_3_N and Ni_3_N–Co_3_N nanoparticles, LSV was performed which can be ascribed to glucose oxidation by these metal nitride catalysts. LSV curves slopes (Fig. 6A) were found ascending in the following manner: Co_3_N < Ni_3_N < Ni_3_N–Co_3_N. A peak current density of 28.3 mA cm^−2^ was obtained at −0.4 V *vs.* Hg/HgO in this work which is almost 1.39 times higher than that of Co_3_N (11.81 mA cm^−2^) and 0.45 times higher than of Ni_3_N (19.42 mA cm^−2^). The obtained current density of bimetallic catalyst Ni_3_N–Co_3_N is higher as compared to mono metal nitride catalysts because the electronic environment of the metal surface is changed by the formation of a heteroatom bond (Fig. 5). That modification improved its electronic structure through the ligand effect, and the geometry of the bimetallic structure also transformed from that of parent metals, resulting in a strain effect that modifies the electronic structure by variation in the orbital overlap.^46^ Secondly, the nitride doping improved its electrical conductivity and catalytic ability. Owing these reasons, the current density of Ni_3_N–Co_3_N is much higher than that of a previous work done on Ni_4_–Co_2_/AC composite catalyst 21.03 mA cm^−2^ (ref. 13) at −0.4 V *vs.* Hg/HgO. Cao *et al.*^47^ suggested that the presence of nitrogen can considerably improve the electronic properties of metals by increasing electrons density on its surface. Wang *et al.*^48^ also declared that the addition of an appropriate amount of nitride could remarkably enhance catalyst performance.

EIS is a powerful diagnostic tool for fuel cells to characterise various limitations and improve fuel cell performance. There are three basic sources of fuel cell voltage loss: charge transfer activation or “kinetic energy” loss, ion and electron transfer or “ohmic” losses, and concentration or “mass transfer” losses. Among other factors, the environmental impact spectrum is an experimental technique that can be used to separate and quantify these polarization sources. Fig. 6B shows the Nyquist plots of all the anodes fitted by using the equivalent electrical circuit method.^49^ The total resistance (*R*_t_) in the equivalent circuit is mainly comprised of three resistances: ohmic resistance (*R*_s_), diffusion resistance (*R*_d_) and charge transfer resistance (*R*_ct_).^38,50^ The fitted EIS data are listed in Table 1. *R*_t_ values declined in the following order: Co_3_N > Ni_3_N > Ni_3_N–Co_3_N. The Ni_3_N–Co_3_N shows the lowest *R*_t_ values compared to Ni_3_N and Co_3_N which are 3.9356 Ω, 4.5438 Ω, 6.682 Ω, respectively.

**Table 1:** Fitted EIS data for different anodes by equivalent electrical circuit

Anode	*R*_s_ (Ω)	*R*_ct_ (Ω)	*R*_d_ (Ω)	*R*_t_ (Ω)
Ni_3_N–Co_3_N	0.6985	0.2671	2.97	3.9356
Ni_3_N	0.9758	1.056	2.008	4.5438
Co_3_N	0.772	1.093	4.817	6.682

To further demonstrate the performance of these electrocatalysts in actual operating fuel cells, a full glucose fuel cell was assembled. Different anodes containing 5% catalysts and a same Cu_2_O–Cu air–cathode were employed to investigate the power density and polarization curves. The performance of full glucose fuel was examined by varying resistance from 9000 to 10 Ω. Fig. 6C depicts the current density and power density curves of these anodes in glucose fuel cells. The fuel cell equipped with the Ni_3_N–Co_3_N anode exhibits a maximum power density of 30.89 W m^−2^ which is almost 2.6 times greater than that of bare AC anode (11.94 W m^−2^), 1.7 times greater than Co_3_N anode (17.47 W m^−2^), and 1.3 times higher than Ni_3_N anode (23.62 W m^−2^). While, the current density of 97.66 A m^−2^, 86.30 A m^−2^, 73.81 A m^−2^, and 58.42 A m^−2^ were obtained in fuel cells equipped with Ni_3_N–Co_3_N, Ni_3_N, Co_3_N, and bare AC, respectively.

The obtained power density of Ni_3_N–Co_3_N is higher than that of reported noble and transitional metals like, Ni_4_–Co_2_/AC (23.97 W m^−2^),^13^ Ag (20.3 W m^−2^),^51^ Pd–Pt/GO/Ni (12.5 W m^−2^)^52^ and Au MnO_2_/C (11 W m^−2^),^53^ correspondingly. However, the potentials of cathodes remained unchanged as shown in Fig. 6D, which reveals that variation in power density is mainly ascribed to the modification in anodes. The fuel cell equipped with the low-cost Ni_3_N–Co_3_N catalyst noticeably has higher power density. It can be supposed that the interactive property of Ni and Co and the presence of nitrogen in the composite boost up the electro-catalytic activity. It has been reported that a covalent bond between metals and nitrogen in metal nitrides and the number of unpaired d-electron offered for intra-bond polarization makes the metals be easily reduced.^54^

## 4. Conclusion

In conclusion, we described a homogeneous 3D-porous structured Ni_3_N–Co_3_N as a promising material for glucose fuel cell. Its unique conductivity and nanostructure are beneficial to redox-related mass transfer reactions. Given the obvious advantages associated with the introduction of nitrogen into a bimetallic catalyst, there is great promise for future support based on nickel–cobalt catalysts. The inclusion of nitride supports the inherited advantage of robust Ni_3_N–Co_3_N, which offer an excellent synergistic effect, the strong metal-support interactions, which enhance the electrical conductivity of the composite. A Glucose fuel cell equipped with Ni_3_N–Co_3_N composite material exhibited a maximum power density of 30.89 W m^−2^, which is higher than that of Ni_3_N, Co_3_N, respectively. These findings would promote the low-cost transition bimetallic nitrides as advanced materials in the electro-catalysis field of glucose direct fuel cells.

## Conflicts of interest

There are no conflicts of interest to declare.

## Supplementary Material
